# Restoring the CD226 in CD8^+^T cells overcomes TIGIT-refractory immunity in HER2^+^ breast cancer

**DOI:** 10.1038/s41419-026-09004-5

**Published:** 2026-07-13

**Authors:** Liyi Zhang, Jiaoduan Li, Bingqiu Xiu, Chen Zheng, Qi Zhang, Huixin Lu, Zehao Wang, Zhibo Shao, Jingyan Xue, Yayun Chi, Dongxi Xiang, Jiong Wu

**Affiliations:** 1 Department of Breast Surgery, Key Laboratory of Breast Cancer in Shanghai, Fudan University Shanghai Cancer Center, Shanghai, PR China; 2 Department of Breast Surgery, the First Affiliated Hospital of Xiamen University, School of Medicine, Xiamen University, Xiamen, PR China; 3 Department of Dermatology, the First Affiliated Hospital of Xiamen University, School of Medicine, Xiamen University, Xiamen, PR China; 4 Department of Oncology, Fudan University Shanghai Medical College, Shanghai, PR China; 5 State Key Laboratory of Systems Medicine for Cancer, Shanghai Cancer Institute, Department of Biliary-Pancreatic Surgery, Department of Gastrointestinal Surgery, Shanghai Key Laboratory of Cancer System Regulation and Clinical Translation, Renji Hospital, Shanghai Jiao Tong University School of Medicine, Shanghai, PR China; 6 Department of Breast Surgery, Fudan University Shanghai Cancer Center Xiamen Hospital, Xiamen, PR China; 7 Collaborative Innovation Center for Cancer Medicine, Shanghai Medical College, Fudan University, Shanghai, PR China

**Keywords:** Breast cancer, Immunization

## Abstract

Immune checkpoint blockade (ICB) has shown limited activity in HER2^+^ breast cancer, yet the mechanisms underlying this refractoriness remain unclear. Integrating single-cell transcriptomics from untreated human and murine HER2^+^ tumors with an anti-HER2 non-sensitive mouse model, a neoadjuvant non-pCR patient cohort, functional co-culture assays, and in vivo perturbations, we identify TIGIT signaling from malignant cells (via CD112) to CD8^+^T cells as a dominant immunosuppressive axis. High TIGIT and CD112 expression correlate with poor clinical outcomes and with the enrichment of TIGIT⁺CD8⁺T cells after anti-HER2 therapy. Therapeutically, combining anti-TIGIT with anti-HER2 reprograms the tumor microenvironment, expanding activated CD8^+^ T cells with enhanced effector function, increasing IFN-γ production, and restoring MHC-I on tumor cells. A central mechanistic node is the reinstatement of the costimulatory receptor CD226 on CD8^+^T cells. TIGIT blockade induces CD226, and CD8 dependence is required for efficacy. Neutralizing CD226 abrogates cytotoxicity and IFN-γ secretion. Multiplex tissue analyses further show that intratumoral CD8⁺CD226^+^T-cell density predicts improved overall and disease-free survival in HER2^+^ disease. Collectively, these data reveal that restoring CD226-mediated co-stimulation overcomes TIGIT-refractory immunity and sensitizes HER2^+^ tumors to anti-HER2 therapy, positioning CD226 as both a pharmacodynamic driver and a clinically actionable biomarker for patient selection and response monitoring.

**Figure:**
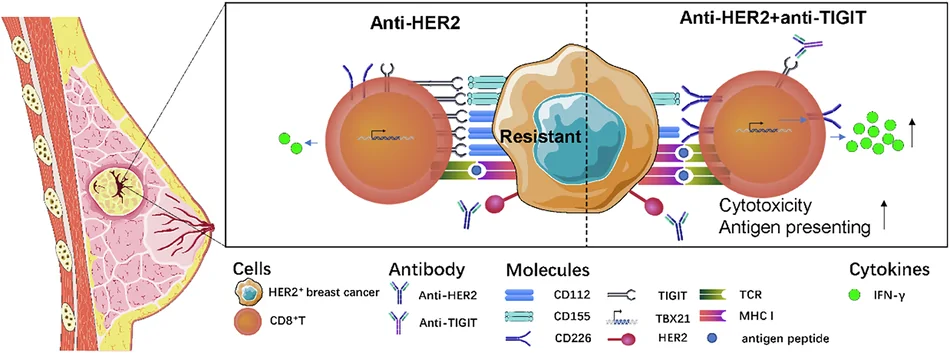
Anti-HER2 therapy fails in HER2⁺ breast cancer due to immunosuppression. This study shows tumor engagement of TIGIT on CD8⁺ T cells suppresses anti-tumor function. Combining anti-TIGIT with anti-HER2 blocks this suppression, reinvigorating CD8⁺ T cells via CD226 and enabling tumor clearance. This combination strategy overcomes therapeutic resistance.

Anti-HER2 therapy fails in HER2⁺ breast cancer due to immunosuppression. This study shows tumor engagement of TIGIT on CD8⁺ T cells suppresses anti-tumor function. Combining anti-TIGIT with anti-HER2 blocks this suppression, reinvigorating CD8⁺ T cells via CD226 and enabling tumor clearance. This combination strategy overcomes therapeutic resistance.

## Introduction

HER2^+^ breast cancer is an aggressive breast cancer subtype. Though HER2-targeting monoclonal antibodies like trastuzumab and pertuzumab and tyrosine kinase inhibitors (TKIs) show positive outcomes, the development of resistance to these drugs has been the major challenges. Emerging evidence suggests that HER2 signaling itself can impair DNA damage repair pathways, potentially contributing to both tumor progression and therapeutic resistance [1]. It has been suggested that HER2^+^ breast cancer shows potential immunotherapeutic potential due to abundant tumor-infiltrating lymphocytes (TILs) in tumor microenvironment (TME) and high tumor mutation burden (TMB) level among breast cancer subtypes [2, 3]. The accumulation of DNA damage may further enhance tumor immunogenicity, theoretically providing a rationale for combining immunotherapy with DNA-targeting agents [4]. Though one humanized mouse tumor-bearing model indicated combination of anti-PD-1 enhanced HER2^+^ breast cancer suppression, limited benefit of anti-PD-1 therapy was found in either early or advanced HER2⁺ breast cancer clinical trials [5–7]. The mechanism by which poor efficacy of anti-PD-1 therapy in HER2^+^ breast cancer is still unclear. Hence, further studies about alternative immune checkpoint blockade to HER2⁺ breast cancer are on urgent.

Single-cell transcriptomic analyses offered further insights into dynamic ecosystem of breast carcinoma cells and diverse cell types including immune cells [8]. Multi-layered crosstalk between malignant and immune cells within TME plays a pivotal role in initiation and progression and therefore offers us an opportunity to uncover potential therapeutic targets against HER2^+^ breast cancer.

TIGIT, a popular inhibitory immune checkpoint, catches eyes on researchers by its blockade demonstrated antitumor activity in many solid tumors [9]. To date, 70 clinical trials has found on ClinicalTrial.gov database among which 37 were phase I, 37 were phase II and 7 were phase III [10]. In pre-clinical studies, TIGIT avoids CD8^+^T immune surveillance in pancreatic cancer, biliary tract cancer, colorectal cancer and neuroblastoma [11–14]. Blockade of TIGIT reversed exhausted natural killer (NK) cells in tumor models including colorectal cancer, triple negative breast cancer (TNBC), melanoma and fibrosarcoma [15, 16]. Nevertheless, given the importance of TIGIT in manipulating an immune response, rare studies about blockade works of TIGIT has found in HER2⁺ breast cancer, a study focusing anti-TIGIT efficacy and TME diversity after anti-TIGIT in HER2⁺ breast cancer might contribute to HER2⁺ breast cancer precise therapy.

Herein, we confirmed TIGIT expression on CD8^+^T cells as a potential therapeutic target in HER2⁺ breast cancer via untreated and anti-HER2 non-sensitive human and mouse samples. We identified improved antitumor immunity by combination anti-HER2 with anti-TIGIT to activate CD8^+^T cells via increased CD226 expression and experimentally revealed that this population was only induced by therapy containing anti-TIGIT. Furthermore, we validated the infiltration of CD8^+^CD226^+^T cells correlated with favorable outcome and proposed CD226 as a candidate biomarker to predict anti-TIGIT efficacy.

## Results

### Re-analysis of untreated HER2^+^ breast cancer TME

**Fig. 1:**
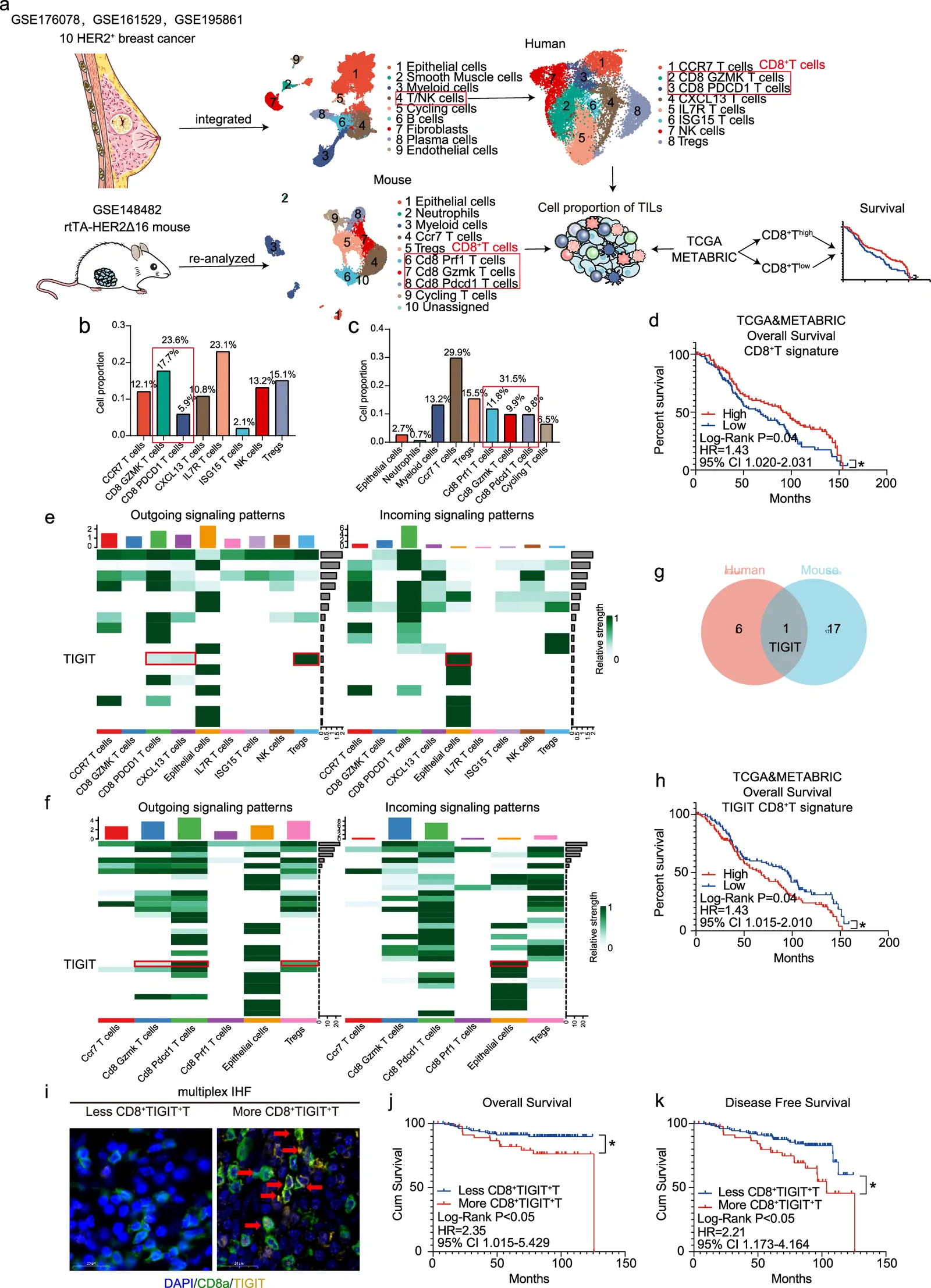
Re-analysis of untreated HER2^+^ breast cancer TIME. **a** Scheme: further analyzed 10 HER2^+^ breast cancer patient samples and a murine HER2^+^ breast cancer model and assessed the proportion of tumor-infiltrating lymphocytes (TILs). Subsequently, survival analysis was performed on HER2^+^ breast cancer patients from the TCGA and METABRIC databases. **b** Cell proportion of TILs in 10 HER2^+^ breast cancer patients. **c** Cell proportion of TILs in a murine HER2^+^ breast cancer model. **d** Kaplan–Meier survival curves of HER2^+^ breast cancer patients from TCGA and METABRIC dataset, stratified by CD8^+^T cells signature. **e** Crosstalk between epithelial cells and immune-infiltrating lymphocytes in HER2^+^ breast cancer patients. **f** Crosstalk between epithelial cells and immune-infiltrating lymphocytes in murine HER2^+^ breast model. **g** The Venn diagram of cell communication results from human and mouse samples. **h** Kaplan–Meier survival curves of HER2^+^ breast cancer patients from TCGA and METABRIC dataset, stratified by TIGIT^+^CD8^+^ T cells signature. Significance levels denote statistically differential expression between groups (*p < 0.05). **i** Representative immunofluorescence micrographs showing variable levels of TIGIT^+^CD8^+^ T cell infiltration in paraffin-embedded tissue sections from Fudan University Shanghai Cancer Center (FUSCC). Left panel: Less TIGIT^+^CD8^+^ T cells. Right panel: More TIGIT^+^CD8^+^ T cells. Nuclei are stained with DAPI (blue). CD8a is labeled in green, and TIGIT is labeled in yellow. Arrows indicate examples of double-positive (TIGIT^+^CD8^+^) T cells. Scale bar: 20 μm. **j** Overall survival curves of 200 HER2^+^ breast cancer patients from FUSCC, stratified by TIGIT^+^CD8^+^ T cells. Significance levels denote statistically differential expression between groups (*p < 0.05). **k** Disease free survival curves of 200 HER2^+^ breast cancer patients from FUSCC, stratified by TIGIT^+^CD8^+^ T cells. Significance levels denote statistically differential expression between groups (*p < 0.05).

To clarify tumor microenvironment (TME) of untreated HER2^+^ breast cancer, we first re-analyzed the single-cell transcriptomes of 10 samples from patients with HER2^+^ breast cancer [17–19]. After quality filtering, the 54,635 cells were analyzed and identified nine embedded clusters including epithelial cells, smooth muscle cells, myeloid cells, T/NK cells, cycling cells, B cells, fibroblasts, plasma cells and endothelial cells in uniform manifold approximation and projection (UMAP) plot with annotation (Fig. 1a and Supplementary Fig. S1a). 13,685 T/NK cells were then divided into eight clusters including *CCR7* T cells, *CD8 GZMK* T cells, *CD8 PDCD1* T cells, *CXCL13* T cells, *IL7R* T cells, NK cells and Tregs with annotation (Fig. 1a and Supplementary Fig. S1b). We then analyzed the proportion of re-clustered T/NK cells. CD8^+^T cells (*CD8 GZMK* T cells and *CD8 PDCD1* T cells) occupied highest proportion (23.6%) in T/NK cluster (Fig. 1b). Next, we reanalyzed 3 untreated tumor-infiltrating lymphocyte (TILs) samples from rtTA-HER2Δ16 mouse [20]. 8115 cells were divided into 10 clusters with annotation after quality control (Fig. 1a, Supplementary Fig. S1c, d). Interestingly, mousy CD8^+^T cells (*Cd8 Prf1* T cells, *Cd8 Gzmk* T cells and *Cd8 Pdcd1* T cells) also showed predominant proportion (31.5%) in TILs, suggesting enriched CD8^+^T cells in HER2^+^ breast cancer TME in murine (Fig. 1c). To further explore the prognostic value of CD8⁺ T cells, we integrated data from the TCGA and METABRIC databases for HER2^+^ breast cancer. We first removed batch effect to obtain integrated data (Supplementary Fig. S1e, f). After stratifying patients based on CD8⁺ T cell infiltration levels, the results demonstrated that high tumor infiltration of CD8⁺ T cells in HER2^+^ breast cancer is associated with a more favorable prognosis (Fig. 1d). Cell-cell communication reveals TIGIT signal as the only common signal between epithelial cells and inhibitory CD8^+^T cells in both human and mouse samples (Fig. 1e–g).

We then focused on the prognostic value of TIGIT in HER2^+^ breast cancer. Despite a lower overall survival trend, TIGIT expression show no significance with prognosis (Supplementary Fig. S1g). Considering TIGIT mostly expressed on immune cells, we used TIGIT^+^CD8^+^T signature to calculate prognosis [21]. Higher TIGIT^+^CD8^+^T signature were associated with poor prognosis in HER2^+^ breast cancer (Supplementary Fig. 1h). 200-tissue microarrays multiplex IHF also revealed more TIGIT^+^CD8^+^T cells infiltration was associated with poor overall survival (OS) and disease-free survival (DFS) in HER2^+^ breast cancer (Fig. 1i–k). Collectively, these data indicated a specific CD8^+^T enriched microenvironment and identified TIGIT^+^CD8^+^ T cells interlinking to poor outcome in HER2^+^ breast cancer.

### Characteristic of TIGIT-CD112/CD155 in HER2/Neu non-sensitive population

**Fig. 2:**
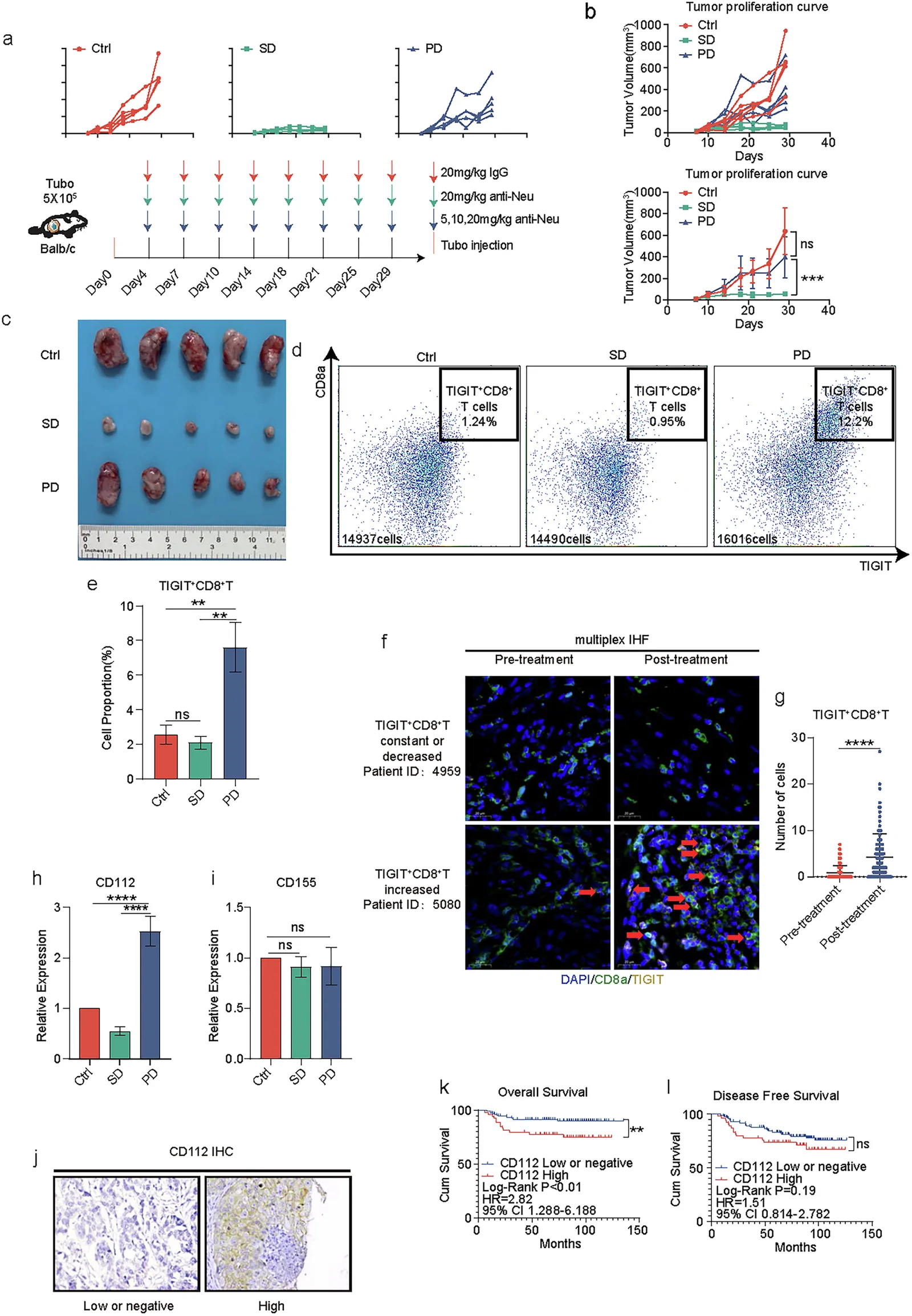
Characteristic of TIGIT-CD112/CD155 in HER2/Neu non-sensitive population. **a** Schematic of establishing anti-Neu resistant murine model: Tubo cells (5 × 10⁵) injected into BALB/c mice. For PD group, Tubo cells injected in day 0 and mice receiving 5 mg/kg anti-Neu by intraperitoneal injection at day 4,7 and 10; 10 mg/kg anti-Neu at day 14, 18 and 21; 20 mg/kg anti-Neu at day 25 and 29. PD was defined as when tumor volume decreased at day 25 and increased again at day 29. For ctrl and SD group, 20 mg/kg isotype IgG and anti-Neu were injected by intraperitoneal injection at day 18, 21, 25 and 29 in ctrl group and sensitive group respectively. **b** Tumor proliferation curve showed in three groups (day 29 mean volume: Ctrl:636.81 mm^3^, SD:57.99 mm^3^, PD:396.62 mm^3^). ns indicates no statistically significant difference while asterisks denote a significant difference (***p < 0.001). **c** Tumor images across three experimental groups. (Top to bottom: Ctrl, SD, and PD). **d** Proportion of tumor-infiltrating TIGIT^+^CD8^+^T cells across three groups. (Left to right: Ctrl, SD, and PD). Data are derived from flow cytometric analysis of dissociated tumor tissues. **e** Statistical analysis of tumor-infiltrating TIGIT^+^CD8^+^T cell proportions across three experimental groups. ns indicates no statistically significant difference, while ** denotes a significant difference (*p* < 0.01). **f** Representative images from 27 patients treated at FUSCC are shown. Nuclei are stained with DAPI (blue), CD8a⁺T cells in green, and TIGIT⁺ cells in yellow. Eight patients (29.6%) showed reduced or unchanged TIGIT^+^CD8^+^T cell densities after treatment. Nineteen patients (70.4%) exhibited increased infiltration of TIGIT^+^CD8^+^T cells post-treatment. **g** Change in the average number of TIGIT^+^CD8^+^T cells per field before and after treatment (paired test). Pre-treatment (0.85 cells) and post-treatment (4.26 cells). Asterisks denote a significant difference (**p < 0.01). Scale bar: 20 μm. **h** Relative *CD112* mRNA expression levels across three experimental groups. (Left to right: Ctrl, SD, and PD, ****p < 0.0001). **i** Relative *CD155* mRNA expression levels across three experimental groups. (Left to right: Ctrl, SD, and PD). ns indicates no statistically significant difference. **j** Representative images of CD112 IHC staining from FUSCC (*n* = 200). Left: classified as low or negative CD112 expression. Right: classified as high CD112 expression. **k** Overall survival curves of 200 HER2^+^ breast cancer patients from FUSCC, stratified by CD112 expression. **l** Disease free survival curves of 200 HER2^+^ breast cancer patients from FUSCC, stratified by CD112 expression.

To explore TIGIT expression in treated HER2^+^ breast cancer, we established a HER2/Neu non-sensitive model based on Tubo cells, a cell line overexpressing HER2/Neu (Fig. 2a and Supplementary Fig. S2a). Control (Ctrl) group showed continuously increasing tumor volume, sensitive group showed a stable volume under high dose (20 mg/kg) of anti-Neu mab and non-sensitive group showed gradually growing tumor under low dose (5 mg/kg), median dose (10 mg/kg) and high dose (20 mg/kg) of anti-Neu mab, respectively (Fig. 2a–c). Flow cytometry showed upregulated TIGIT^+^CD8^+^T cells in non-sensitive group comparing with ctrl and sensitive group (Fig. 2d, e and Supplementary Fig. S2b). Notably, neither CD8^+^PD-1^+^T cells nor TIGIT^+^NK cells showed significant discrepancy in three groups (Supplementary Fig. S2c–f). These data indicated non-sensitive anti-HER2/Neu treatment correlates with upregulation of TIGIT on CD8^+^T cells. To further confirm this correlation, we then chose a cohort in FUSCC containing 27 non-pCR patients with HER2⁺ breast cancer undergoing neoadjuvant therapy as non-sensitive population. Analysis of paired patient samples (pre- vs. post-treatment) by multiplex immunofluorescence revealed a significant increase in the density of TIGIT⁺CD8⁺ T cells following therapy (Fig. 2f, g). Considering CD112/CD155 were main ligands of TIGIT on malignant cells, we then explored whether CD112/CD155 change or not after anti-HER2/Neu therapy [22]. *CD112* mRNA expression in HER2/Neu non-sensitive model was upregulated in non-sensitive group comparing with ctrl and sensitive groups while *CD155* mRNA expression showed no difference in three groups (Fig. 2h, i). IHC staining of tumor tissues from 27 breast cancer patients revealed upregulated CD112 expression in post-treatment rather than pre-treatment whereas CD155 expression showed no significant difference between both groups (Supplementary Fig. S3a–d). The 200-tissue microarray verified high CD112 expression correlated with poor OS whereas no statistical difference with DFS (Fig. 2j–l and Table 1). Besides, IHC staining of CD155 expression in 200 tissue microarrays showed high CD155 expression was associated with poor OS and DFS (Supplementary Fig. S3e–g and Table 2). These data indicated TIGIT correlated with HER2/Neu non-sensitive population and suggesting TIGIT as a potential therapeutic target in HER2^+^ breast cancer.

**Table 1:** CD112 expression and clinical characteristics.

Variable	CD112 expression		Patients,No.	Pearson	*P* value
	Low or not	High			
Age,y				0.323	0.354
≤60	116 (71.6%)	46 (28.4%)	162		
>60	22 (66.7%)	11 (33.3%)	33		
Histological grade				2.923	0.404
II	78 (72.9%)	29 (27.1%)	107		
III	58 (67.4%)	28 (32.6%)	86		
TNM stage				29.380	<0.001^***^
I	21 (72.4%)	8 (27.6%)	29		
II	92 (83.6%)	18 (16.4%)	110		
III	23 (42.6%)	31 (57.4%)	54		
Ki67				12.139	<0.01^**^
≤20%	55 (82.1%)	12 (17.9%)	67		
>20%	38 (54.3%)	32 (45.7%)	70		
LR status				0.151	0.473
No	126 (70.4%)	53 (29.6%)	179		
Yes	12 (75.0%)	4 (25.0%)	16		
DM status				0.075	0.467
No	116 (71.2%)	47 (28.8%)	163		
Yes	22 (68.8%)	10 (31.2%)	32		

Table 1 CD112 expression and clinical characteristics of 200 patients from FUSCC.

*LR* Local recurrence, *DM* Distant metastasis.

**Table 2:** CD155 expression and clinical characteristics.

Variable	CD155 expression		Patients,No.	Pearson	*P* value
	Low or not	High			
Age,y				1.387	0.165
≤60	115 (71.0%)	47 (29.0%)	162		
>60	20 (60.6%)	13 (39.4%)	33		
Histological grade				4.102	0.251
II	79 (73.8%)	28 (26.2%)	107		
III	55 (64.0%)	31 (36.0%)	86		
TNM stage				21.569	<0.001^***^
I	21 (72.4%)	8 (27.6%)	29		
II	88 (80.0%)	22 (20.0%)	110		
III	24 (44.4%)	30 (55.6%)	54		
Ki67				3.320	0.073
≤20%	50 (74.6%)	17 (25.4%)	67		
>20%	42 (60.0%)	28 (40.0%)	70		
LR status				1.379	0.264
No	126 (70.4%)	53 (29.6%)	179		
Yes	9 (56.2%)	7 (43.8%)	16		
DM status				8.982	<0.01^**^
No	120 (73.6%)	43 (26.4%)	163		
Yes	15 (46.9%)	17 (53.1%)	32		

Table 2 CD155 expression and clinical characteristics of 200 patients from FUSCC.

*LR* Local recurrence, *DR* Distant metastasis.

### TIGIT/HER2 co-blockade restrains tumors and restores anti-Neu sensitivity of resistant subclones in the TME

**Fig. 3:**
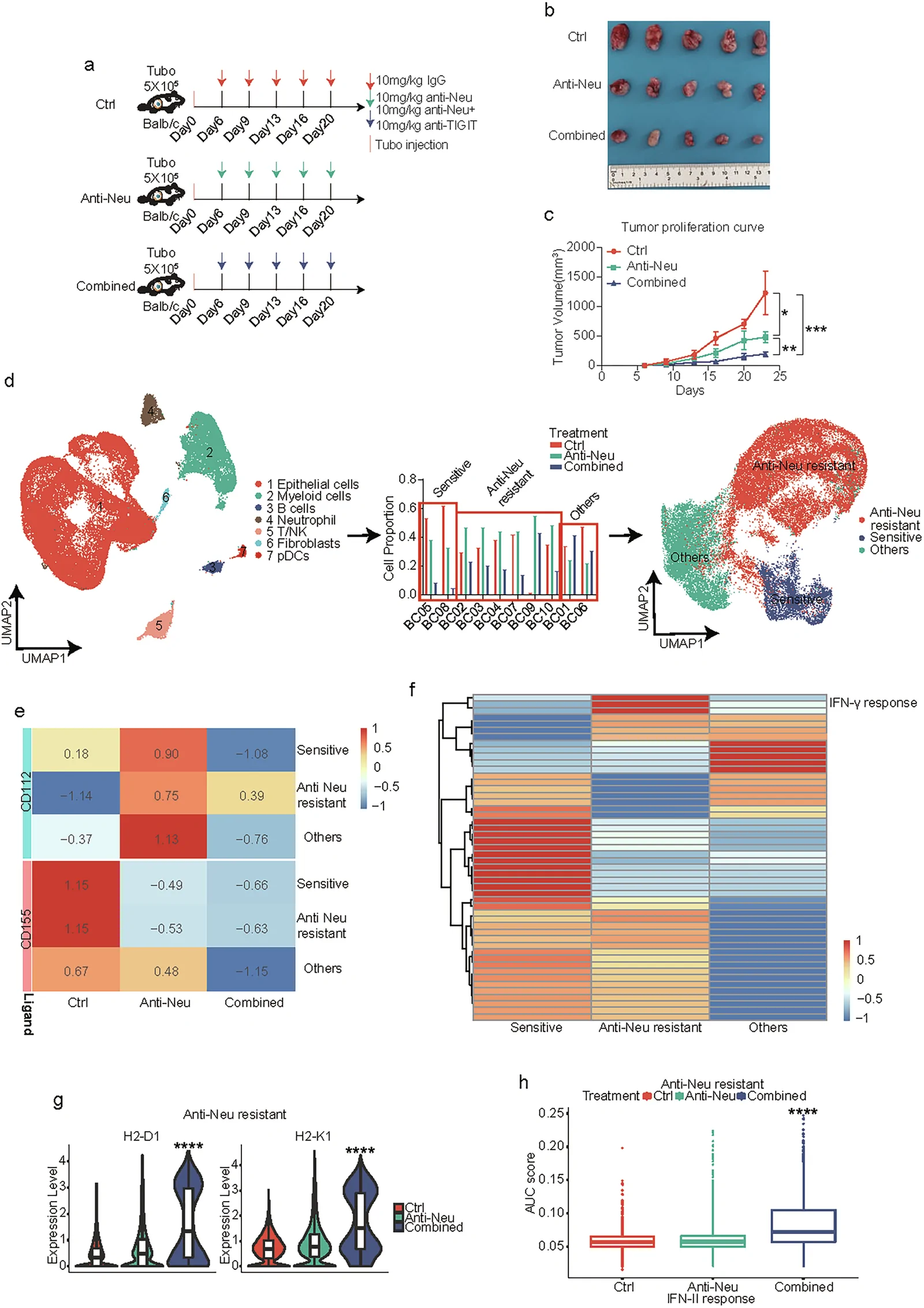
TIGIT/HER2 co-blockade restrains tumors and restores anti-Neu sensitivity of resistant subclones in the TIME. **a** Schematic of in vivo study: Tubo cells (5 × 10⁵) injected into BALB/c mice. Mice received IgG isotype (10 mg/kg), anti-Neu mab (10 mg/kg) or combination of anti-Neu mab (10 mg/kg) and anti-TIGIT mab (10 mg/kg) twice weekly. **b** Tumor images across three experimental groups. (Top to bottom: Ctrl, Anti-Neu, and Combined). **c** Tumor proliferation curve showed in three groups (day 23 mean volume: Ctrl:1233.21 mm^3^, Anti-Neu:483.58 mm^3^, Combined:200.43 mm^3^), whereas asterisks denote a significant difference (*p < 0.05, **p < 0.01, ***p < 0.001). **d** UMAP projection displaying the tumor components of seven individual cells. Malignant cells were re-clustered into 10 distinct subpopulations and categorized to sensitive subclones, anti-Neu resistant subclones and others. **e** scRNA-seq analysis reveals *CD112* and *CD155* mRNA expression across three treatment groups. (Top:*CD112* bottom:*CD155*). **f** GSVA scores of HALLMARK gene sets across three experimental groups. (Left to right: sensitive subclones, anti-Neu resistant subclones, and others). **g** scRNA-seq analysis of anti-Neu-resistant tumor subclones treated with Control, anti-Neu monotherapy, or Combination therapy. Violin plots depict normalized expression levels of H2-D1 and H2-K1, key MHC class I genes involved in antigen presentation and immune evasion. Asterisks determine a significant difference (****p < 0.0001). **h** scRNA-seq data from anti-Neu-resistant subclones treated with Control, anti-Neu monotherapy, or Combination therapy were analyzed via AUCell package. The AUC score quantifies the activity of the IFN-II response gene signature. Asterisks determine a significant difference (****p < 0.0001).

We then evaluate whether TIGIT can be an effective target in HER2^+^ breast cancer in vivo. Briefly, mice were treated via intraperitoneal injection with IgG isotype (ctrl group), anti-Neu mab, and a combination of anti-Neu mab and anti-TIGIT mab (Fig. 3a). Tumor volumes were significantly reduced in combined group comparing with ctrl, anti-Neu and anti-TIGIT group (Fig. 3b, c and Supplementary Fig. S4a). Samples were then harvested and subjected to single-cell RNA sequencing (scRNA-seq). 47,996 cells were divided into seven clusters with annotation after quality filtering (Supplementary Fig. S4b). Malignant cells were then divided into ten subclusters (Supplementary Fig. S4c). Next, we defined three subclones. Sensitive subclones exhibited sequentially decreased proportions in the ctrl, Anti-Neu group, and Combined group; anti-Neu-resistant subclones showed increased proportions in the Anti-Neu group compared to both Control and Combined groups; Others included subclones with unchanged or non-consistent proportional changes. (Fig. 3d and Supplementary Fig. S4c). *CD112* mRNA expression was increased in anti-Neu group and reduced in combined group, whereas *CD155* mRNA expression were reduced both in anti-Neu group and combined group, suggesting a downregulated malignant potential of malignant cells after combination therapy (Fig. 3e).

Gene set variation analysis (GSVA) analysis of HALLMARK gene sets indicated highest IFN-γ response pathway in anti-Neu resistant subclone (Fig. 3f), suggesting anti-Neu resistant subclone was associated with antigen presenting [23]. We then evaluated MHC-I associated gene *H2-D1* and *H2-K1* in anti-Neu resistant subclone. *H2-D1* and *H2-K1* showed highest expression in combined group comparing with ctrl and anti-Neu groups (Fig. 3g). Gene set activity analysis also indicated highest IFN-II response scores in combined group (Fig. 3h and Supplementary Fig. S4d). These results verified the efficacy of combination anti-TIGIT with anti-HER2/Neu and indicated more sensitive anti-Neu resistant subclone after combination therapy.

### Combination therapy promoted CD8^+^T infiltration and activation in TME

**Fig. 4:**
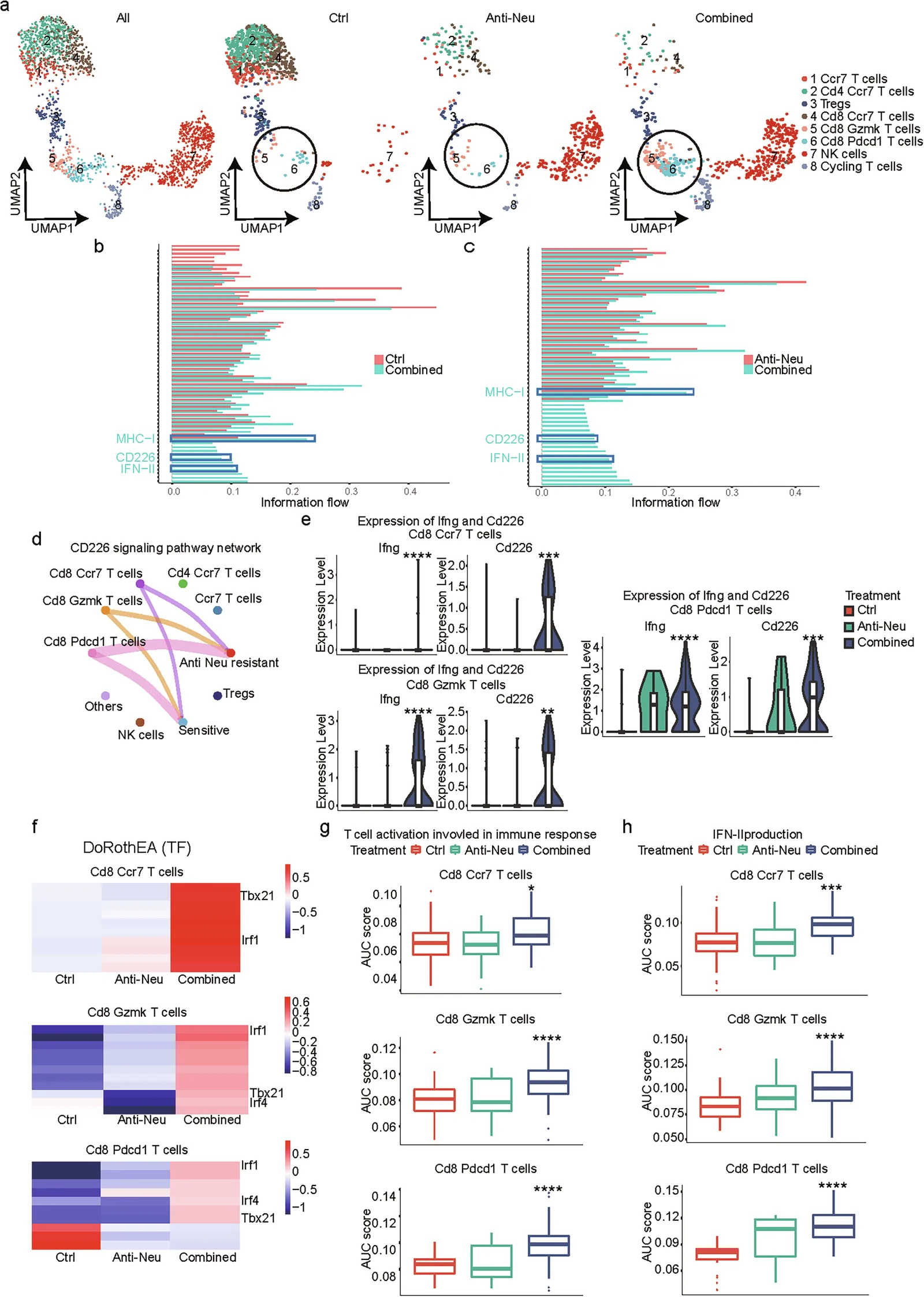
Combination therapy promoted CD8^+^T infiltration and activation in TIME. **a** UMAP plot displaying the T/NK cells in three groups. *Cd8 Gzmk* T cells (Ctrl: 28.48%, Anti-Neu: 15.23%, Combined: 56.29%); *Cd8 Pdcd1* T cells (Ctrl: 15.87%, Anti-Neu: 6.35%, Combined: 77.78%). **b** Differential intercellular communication between Combined Treatment and Control groups. Signaling edges colored in red represent interactions stronger in the Control group, while green edges indicate interactions enhanced in the Combined group. Labels in green highlight specific signals (CD226, MHC-I, and IFN-II response) significantly strengthened in the Combined group. **c** Differential intercellular communication between Anti-Neu Treatment and Combined groups. Signaling edges colored in red represent interactions stronger in the Anti-Neu group, while green edges indicate interactions enhanced in the Combined group. Labels in green highlight specific signals (CD226, MHC-I, and IFN-II response) significantly strengthened in the Combined group. **d** Cell communication of CD226 signaling between CD8⁺T cell subsets and tumor subclones. Edge thickness represents the inferred interaction strength, and arrows indicate the direction of signaling from sender to receiver cells. CD226 signaling is primarily emitted from CD8⁺T cell subsets, with the strongest signal originating from *Cd8 Pdcd1* T cells, followed by *Cd8 Gzmk* T cells, and then *Cd8 Ccr7* T cells. These signals are received by both sensitive subclones and anti-Neu-resistant subclones. **e** Expression of Ifng and Cd226 in CD8⁺T cell subsets across three treatment groups. (top to bottom: *Cd8 Ccr7* T cells, *Cd8 Gzmk* T cells, and *Cd8 Pdcd1* T cells). scRNA-seq shows normalized gene expression levels of *Ifng* and *Cd226*. Data are represented for the Control group (red), anti-Neu monotherapy group (green), and Combination therapy group (blue). Significance levels denote statistically differential expression between groups (**p < 0.01, ***p < 0.001, ****p < 0.0001). **f** Analysis of transcription factor activity changes in three CD8⁺ T cell subsets (top to bottom: *Cd8 Ccr7* T cells, *Cd8 Gzmk* T cells, and *Cd8 Pdcd1* T cells) in Tubo mouse tumor model following treatment using the DoRothEA package. **g** AUC scores of T cell activation involved in immune response across CD8⁺T cell subsets under three treatment conditions. scRNA-seq analysis of CD8⁺T cell subsets (top to bottom: *Cd8 Ccr7* T cells, *Cd8 Gzmk* T cells, and *Cd8 Pdcd1* T cells) treated with Control (red), anti-Neu monotherapy (green), or Combination therapy (blue). AUC scores quantify the activity of the T cell activation involved in immune response gene signature. Significance levels denote statistically differential expression between groups (*p < 0.05, ****p < 0.0001). **h** AUC scores of IFN-II production in CD8⁺T cell subsets across three treatment conditions. scRNA-seq analysis of CD8⁺T cell subsets (top to bottom: *Cd8 Ccr7* T cells, *Cd8 Gzmk* T cells, and *Cd8 Pdcd1* T cells) treated with Control (red), anti-Neu monotherapy (green), or Combination therapy (blue). AUC scores quantify the activity of the IFN-II production gene signature. Significance levels denote statistically differential expression between groups (***p < 0.001, ****p < 0.0001).

As more sensitive tumor cells in TME resembled part of efficacy and TIGIT showed effort on T/NK cells according to previous studies, we then focus on T/NK cells in TME [22]. T/NK cells were divided into eight clusters with annotation (Fig. 4a and Supplementary Fig. S4e). An increasing ratio of *Gzmk*^+^ and *Pdcd1*^+^ Cd8^+^T cells was found in the combined group, indicating more CD8^+^T cells infiltrated and pivotal roles of these cells made in combination therapy (Fig. 4a and Supplementary Fig. S4f). Moreover, we re-analyzed and annotated scRNA-seq data about using 4D5 (anti-HER2 mab) in MM3MG-HER2Δ16 cell-bearing mice to further validate results in ctrl and anti-Neu groups in our research [24] (Supplementary Figs. S4g, h, S5a–f).

Cell-cell communication about malignant cells and T/NK cells showed upregulated immune-related signals MHC-I, CD226 and IFN-II when comparing the combined group with ctrl and anti-Neu groups (Fig. 4b, c). Neither anti-Neu group nor 4D5 group presented their increase while comparing with ctrl group (Supplementary Fig. S6a, b). Specifically, beyond the dominant CD226 signals, our analysis revealed that several other cell-cell communication pathways including CCL, MHC-II, CX3C, CDH, GRN, LCK, APP, MIF and ITGAL-ITGB2 were significantly strengthened in the combination treatment group (Fig. 4b, c). Singal pathway network resembled CD226 signal was emitted only by CD8^+^T cells (*Cd8 Ccr7* T cells, *Cd8 Gzmk* T cells and *Cd8 Pdcd1* T cells) and received by sensitive and anti-Neu resistant subclones (Fig. 4d). *Ifng* and *Cd226* mRNA expression were also increased in three types of CD8^+^T cells in TME (Fig. 4e). Interestingly, *Pdcd1* expression on CD8^+^T cells in neither our dataset or public dataset performed no statistical difference in either anti-HER2 or combination of anti-TIGIT and anti-HER2 (Supplementary Fig. S6c, d), which may elucidate why anti-PD-1 therapy showed negative results in HER2⁺ breast cancer PANACEA and IMpassion050 trials to some extent [5, 6]. Transcription factor (TF) analysis found increased IFN-γ related TF *Irf1* and *Irf4*, and CD226-related TF *Tbx21* in three types of CD8^+^T cells in TME via DoRothEA package, suggesting a potential mechanism whereby *TBX21* activation enhances CD8⁺T cell function by promoting CD226-mediated co-stimulation (Fig. 4f). We extended our investigation on MHC class I-mediated signaling across different treatment groups. Ligand–receptor analysis revealed a robust reinforcement of MHC-I–CD8 signaling under therapy. While ctrl tumors were dominated by *H2d1-* and *H2k1-Cd8b1*, anti-Neu therapy introduced the high-affinity *H2d1-Cd8a* interaction. Strikingly, the combined regimen consolidated the strongest axes, *H2d1-* and *H2k1- CD8b1*, indicating strengthened antigen presentation and sustained CD8⁺T-cell engagement. Notably, none of these pairs exhibited significant signaling communication between NK cells and tumor cells in any treatment condition (Supplementary Fig. S6e–g). Gene set activity analysis revealed upregulated T cell activation and IFN-II production pathway scores in combined groups in three types of CD8^+^T cells in TME via AUCell package (Fig. 4g, h, Supplementary Fig. S7a, b). These data suggested CD8^+^T cells in TME were recruited and promoted by combination therapy.

### Combination efficacy is CD8^+^T-cell-dependent and requires CD226 upregulation in vitro and in vivo

**Fig. 5:**
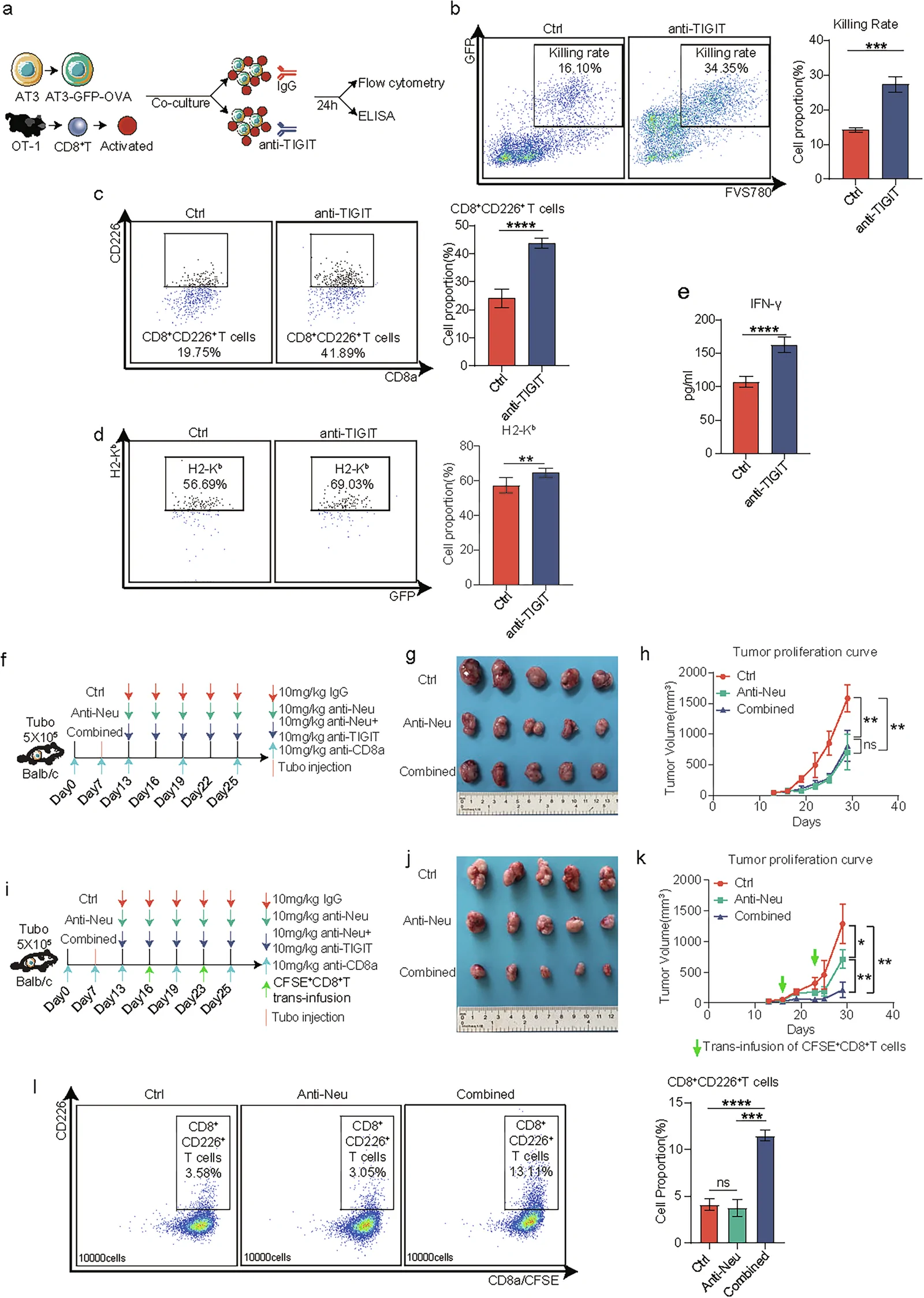
Combination efficacy is CD8^+^ T-cell-dependent and requires CD226 upregulation in vitro and in vivo. **a** Schematic of in vitro study. **b** (Left) Tumor cell killing capacity in the anti-TIGIT group (mean killing: 27.32%) and the Control group (mean killing: 14.15%). (Right) Statistical significance was determined by unpaired Student’s *t* test (***p < 0.001). **c** (Left) Representative flow cytometry plots shows the proportion of CD8⁺CD226⁺ T cells in the anti-TIGIT group (mean: 43.75%) and the Control group (mean: 24.11%). (Right) Statistical significance was determined by unpaired Student’s *t* test (****p < 0.0001). **d** (Left) Representative flow cytometry plots shows the proportion of H2-K^b^-positive tumor cells in the anti-TIGIT group (mean: 64.6%) and the Control group (mean: 57.47%). (Right) Statistical significance was determined by unpaired Student’s *t* test (**p < 0.01). **e** IFN-γ levels in the anti-TIGIT group (mean: 162.32 pg/ml) and the Control group (mean: 107.20 pg/ml). Statistical significance was determined by unpaired Student’s *t* test (****p < 0.0001). **f** Schematic of CD8^+^T depletion treatment: mice receiving anti-CD8a mab at day0, 7, 13, 19 and 25. This regimen was applied to all mice in the designated experiments. 5 × 10^5^ Tubo were implant in Balb/c mice at fourth mammary pad at day7; 10 mg/kg isotype IgG, 10 mg/kg anti-Neu, 10 mg/kg anti-Neu and 10 mg/kg anti-TIGIT were injected by intraperitoneal injection at day13, 16, 19, 22 and 25 in ctrl group, anti-Neu group and combined group respectively. **g** Tumor images across three experimental groups. (Top to bottom: Ctrl, Anti-Neu, and Combined). **h** Tumor proliferation curve showed in three groups (day 29 mean volume: Ctrl: 1582.26 mm^3^, Anti-Neu: 706.78 mm^3^, Combined: 809.80 mm^3^), whereas asterisks denote a significant difference (ns indicates no statistically significant difference, **p < 0.01). **i** Schematic of adoptive infusion treatment: mice underwent the same anti-CD8a depletion schedule. Subsequently, 5 × 10^5^ CFSE-labeled CD8^+^T cells were injected by tail vein at day 16 and day23. Tumor treatment schedules remained identical to the CD8 depletion treatment. **j** Tumor images across three experimental groups. (Top to bottom: Ctrl, Anti-Neu, and Combined). **k** Tumor proliferation curve showed in three groups (day 29 mean volume: Ctrl:1284.26 mm^3^, Anti-Neu:712.19 mm^3^, Combined:211.21 mm^3^), whereas asterisks denote a significant difference (*p < 0.05, **p < 0.01). **l** Proportion of CD8⁺CD226⁺ T cells post-adoptive transfer across three treatment groups. (Left) Representative flow cytometry plots demonstrates the proportion of CD8⁺CD226⁺ T cells in the Control (mean: 4.14%), anti-Neu monotherapy (mean: 3.76%), and Combination therapy (mean: 11.51%) groups. (Right) Significance levels denote statistically differential expression between groups (ns indicates no statistically significant difference, ***p < 0.001, ****p < 0.0001).

To validate results in the scRNA-seq data, we built an in vitro system to co-culture malignant cells and CD8^+^T cells (Fig. 5a and Supplementary Fig. S8a). After 24 h of co-culture, flow cytometry analysis showed that the anti-TIGIT group performed a significantly higher killing rate against malignant cells, with more CD226 expression on CD8^+^T cells, improved H2-K^b^ expression on malignant cells, compared to ctrl group (Fig. 5b–d and Supplementary Fig. S8b–d). Supernatant of co-culture was collected and measured via ELISA. Augmented IFN-γ secretion was found in the anti-TIGIT group (Fig. 5e).

Given this efficacy, we next asked whether manipulating CD8^+^T cells in TME could reprogram pro-immune TME. We then verified the efficacy of anti-CD8a mab via intraperitoneal injection and used this mab to block CD8^+^T cells in TME according to the time scheme (Fig. 5f, Supplementary Fig. S8e, f). Though both anti-Neu and combined groups performed less tumor volume compared with ctrl group, no statistical discrepancy between these two groups in the absence of CD8^+^T cells (Fig. 5g, h). These data indicated loss of CD8^+^T cells in TME undermined the efficacy of combination therapy. Next, we trans-infused CFSE-labeled CD8^+^T cells via intravenous injection at day 16 and day 23 in another batch of CD8^+^T-depleted mice to restore CD8^+^T cells in vivo (Fig. 5i and Supplementary Fig. S8g). The efficacy of the combined group was recovered after CD8^+^T trans-infusion (Fig. 5j, k). Tumor samples were then harvested and digested for flow cytometry analysis. Further proportion of CD8^+^CD226^+^ T cells was increased only in combined group, consistent with scRNA-seq results (Fig. 5l). Taken together, these data confirmed the unique immune-regulated role of CD8^+^T cells and upregulated CD226 expression on CD8^+^T cells in TIGIT combination therapy in HER2^+^ breast cancer.

### TIGIT blockade drives CD226-high CD8^+^ IFN-γ secretion, with CD8^+^CD226^+^ cells marking responders

**Fig. 6:**
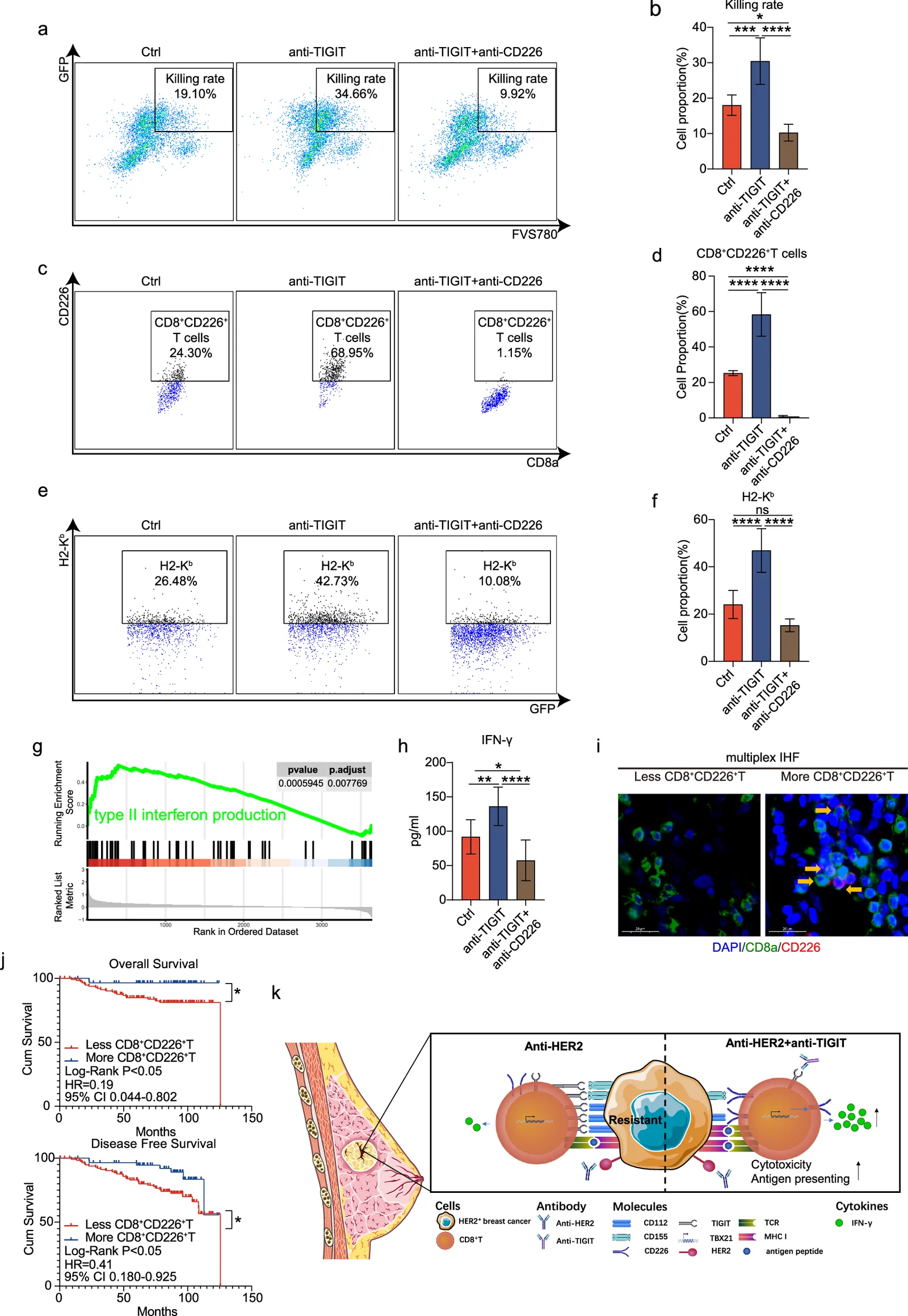
TIGIT blockade drives CD226-high CD8^+^ IFN-γ secretion, with CD8^+^CD226^+^ cells marking responders. **a** Representative flow cytometry plots of tumor cell killing show the cytotoxicity in the Control group (mean: 18.03%), anti-TIGIT monotherapy group (mean: 30.44%), and anti-TIGIT+anti-CD226 combination group (mean: 10.27%). **b** Statistical analysis across three experimental groups (*p < 0.05, ***p < 0.001, ****P < 0.0001). **c** Representative flow cytometry plots show the proportion of CD8⁺CD226⁺ T cells in the Control group (mean: 25.26%), anti-TIGIT monotherapy group (mean: 58.34%), and anti-TIGIT+anti-CD226 combination group (mean: 0.60%). **d** Statistical analysis across three experimental groups (****p < 0.0001). **e** Flow cytometry analysis shows the proportion of H2-K^b^-positive tumor cells in the Control group (mean: 24.07%), anti-Neu monotherapy group (mean: 46.97%), and anti-TIGIT+anti-CD226 combination therapy group (mean: 15.27%). **f** Statistical analysis across three experimental groups (ns indicates no statistically significant difference, ****P < 0.0001). **g** Gene Set Enrichment Analysis (GSEA) of transcriptional profiles between CD226-high and CD226-low CD8⁺T cells (**p < 0.01). **h** ELISA results show IFN-γ levels in the Control group (mean: 91.85 pg/mL), anti-TIGIT monotherapy group (mean: 136.23 pg/mL), and anti-TIGIT+anti-CD226 combination therapy group (mean: 57.53 pg/mL). Statistical analysis across three experimental groups (*p < 0.05, **p < 0.01, ****P < 0.0001). **i** Representative immunofluorescence micrographs showing variable levels of CD8^+^CD226^+^ T cell infiltration in paraffin-embedded tissue sections from FUSCC. Left panel: Less CD8^+^CD226^+^ T cells. Right panel: More CD8^+^CD226^+^ T cells. Nuclei are stained with DAPI (blue). CD8a is labeled in green, and CD226 is labeled in red. Arrows indicate examples of double-positive (CD8^+^CD226^+^) T cells. Scale bar: 20 μm. **j** Overall survival (top) and disease free survival (bottom) curves of 200 HER2^+^ breast cancer patients from FUSCC, stratified by CD8^+^CD226^+^ T cells. Significance levels denote statistically differential expression between groups (*p < 0.05). **k** Graphical abstract illustrating the key mechanistic pathways proposed in this study.

As an increased proportion of CD8^+^CD226^+^ T cells was found in vitro and in vivo, we then tried to decode the value of these cells. Anti-TIGIT group showed the highest killing rate to tumor cells, whereas the anti-TIGIT+anti-CD226 group performed the lowest among three groups (Fig. 6a, b and Supplementary Fig. S9a). After 24 h of co-culture, the proportion of CD8^+^CD226^+^ T cells increased in the anti-TIGIT group, whereas nearly eliminated in the anti-TIGIT+anti-CD226 group, suggesting the efficacy of anti-CD226 mab (Fig. 6c, d and Supplementary Fig. S9b). For MHC-I expression, anti-TIGIT group showed the most H2-K^b^ expression, whereas ctrl group and anti-TIGIT+anti-CD226 group shared similar H2-K^b^ expression (Fig. 6e, f and Supplementary Fig. S9c). To further define the relationship between TBX21 and CD226 at the single-cell level, we performed a secondary clustering analysis on tumor-infiltrating CD8⁺ T cells defined by CD226 expression (high vs. low). Interrogation of *TBX21* expression across these subsets revealed a significant enrichment of *TBX21* within the CD226-high population, indicating that TBX21 is a hallmark of the CD8⁺ T cell state associated with high CD226 co-stimulation (Supplementary Fig. S9d). To functionally validate the role of CD226 in CD8⁺ T cells, we performed differential pathway analysis comparing CD226-high and CD226-low CD8⁺T cell subsets. The results demonstrated that high CD226 expression in CD8⁺T cells effectively promote IFN-γ secretion (Fig. 6g). Consistent with this finding, subsequent ELISA experiments confirmed that CD226-high CD8⁺T cells secreted significantly higher levels of IFN-γ compared to their CD226-low counterparts. To further probe the functional interaction between CD226 and its coinhibitory counterpart TIGIT, we employed a co-culture system consistent with our previous experimental design. The addition of anti-TIGIT antibody enhanced IFN-γ secretion in the co-culture supernatant, suggesting a release of CD8⁺T cells from TIGIT-mediated suppression. Crucially, this enhanced secretion was effectively abolished by the subsequent addition of an anti-CD226 neutralizing antibody, indicating that the observed IFN-γ production is specifically dependent on CD226 signaling (Fig. 6h). Finally, we investigated the predictive value of CD8^+^CD226^+^ T cells in TME. Multiplex IHF staining of 200 tissue microarrays showed that more CD8^+^CD226^+^ T cells infiltration was associated with favorable OS and DFS in HER2^+^ breast cancer (Fig. 6i, j).

To conclude, anti-HER2/Neu mAb therapy is insufficient in HER2⁺ breast cancer owing to compensatory immunosuppression within the TME. Engagement of TIGIT on CD8⁺T cells by its ligands CD112/CD155 on malignant cells imposes a dominant coinhibitory signal that dampens *TBX21* expression and blunts effector differentiation, cytotoxic function, and IFN-γ production. This dysfunctional state is accentuated in anti-HER2–non-sensitive subclones, thereby reinforcing therapeutic resistance. Co-administration of anti-TIGIT mAb with anti-HER2/Neu overcomes this brake by disrupting the CD112/CD155–TIGIT axis, restoring the costimulatory landscape through upregulation of CD226 on CD8⁺T cells, and re-establishing a *TBX21* to CD226 program that potentiates IFN-γ secretion. The resulting reactivation loop enhances MHC-I–restricted tumor recognition and augments CD8⁺T-cell cytotoxicity, yielding effective elimination of malignant cells (Fig. 6k).

## Discussion

Despite failure of PANACEA and IMpassion 050 trails, enriched TILs in HER2⁺ breast cancer TME heralds potential of immunotherapy in HER2^+^ breast cancer [5, 6, 25]. Stimulation of NK cells mediated ADCC is a major mechanism of trastuzumab [26]. Though the presence of CD8^+^T cells is essential for trastuzumab efficacy, the anti-tumor potential of CD8^+^T cells has not been effectively exploited [27, 28]. Considering these specific characteristic, alternative immune therapeutic strategies have been sought. Our study confirmed enriched CD8^+^T cells and present TIGIT signal between CD8^+^T cells and malignant cells in untreated human and mouse HER2^+^ breast cancer TME. Further upregulated TIGIT expression on CD8^+^T cells was found in HER2 non-sensitive population. Combination of anti-HER2/Neu and anti-TIGIT inhibited tumor growth via upregulated CD226 expression on CD8^+^T cells. Enrichment of CD8^+^CD226^+^ T cells could become a biomarker of anti-TIGIT efficacy. Thus, our findings imply a novel therapeutic target to treat HER2^+^ breast cancer.

To delineate the TME of HER2^+^ breast cancer, we re-analyzed the TME in untreated human and mouse at single cell scale. As expected, CD8^+^T cells cluster occupied major cell type in TILs, which coordinated with previous reports on pathological section [25, 29]. Furthermore, TIGIT signal was found between CD8^+^T cells and malignant epithelial cells, suggesting TIGIT as an effective target in HER2^+^ breast cancer. Of note, few signals were emitted from NK cells in human sample, whereas no NK cells were found in mouse samples, maybe because low infiltration and activation of NK cells without anti-HER2 treatment [20, 30, 31]. To mimic HER2 non-sensitive population, we built a HER2 non-sensitive model. We discovered higher TIGIT^+^CD8^+^ T cell proportion in non-sensitive group comparing with ctrl and sensitive group. Consistent with that, increased TIGIT^+^CD8^+^ T cell infiltration was found in HER2 non-pCR patients after standard anti-HER2 treatment. Besides, similar TIGIT^+^NK cell proportion and CD8^+^PD-1^+^T cell proportion were found among three groups, probably because lack TIGIT expression in activated NK cells after anti-HER2 treatment and explained limited anti-PD-1 efficacy in HER2 breast cancer to some extent [32]. Moreover, CD112, a ligand of TIGIT, significantly increased in these HER2 non-sensitive population after treatment whereas no significant change was found on another ligand CD155. The specific upregulation of CD112, but not CD155, hints at a preferential engagement of the TIGIT-CD112 axis in this clinical setting. Similar to the upregulation of CD155 rather than CD112 observed in multiple myeloma, this selectivity may stem from the differential transcriptional regulation of the *CD112* and *CD155* genes under therapy-induced stress signals. Alternatively, it may indicate that CD112 plays a more dominant role in mediating TIGIT-dependent immunosuppression in HER2⁺ breast cancer [33]. CD112 and CD155 were correlated with unfavorable outcome in many solid tumors and we validated them in HER2^+^ breast cancer [13, 34, 35].

Our study also suggested that combination of anti-TIGIT and anti-HER2 significantly inhibited tumor growth. Based on our knowledge, this is the first study that demonstrates combination with anti-TIGIT and anti-HER2 in HER2 breast cancer. We evaluated the efficacy of combination therapy based on tumor cells and immune cells at single cell scale. To further evaluated functions about malignant cells, we divided tumor cells into three subclones according to cell proportion variety after treatment. Reduced *CD112* and *CD155* after combination therapy suggested lower malignant property [36, 37]. Notably, we found highest IFN-γ response pathway in anti-Neu resistant subclone. Considering IFN-γ induced MHC-I expression on tumor cells and thereby promoted tumor apoptosis and recognized by immune cells, we then calculated MHC-I expression and IFN-γ related pathway on anti-Neu resistant subclone [23, 38]. As expected, highest MHC-I expression and IFN-γ response scores were found in combination group, indicated sensitized tumor cells were forming. Therefore, it would be interesting to further evaluate the role of immune cells in TME.

Our study found more effective and exhausted CD8^+^T cells engaged and increased CD226 signal, a more cytotoxic signature of CD8^+^T cells, released from CD8^+^T cells only in combination therapy involved anti-TIGIT [39]. Consistent with that, CD8^+^T cells in combined group showed highest activation and IFN-γ production. Previous studies indicated that type I and II interferon secretion were essential for anti-HER2/Neu but not lymphocyte-mediated cytotoxicity, while anti-HER2 synergized anti-TIGIT in our study fully utilized the CD8^+^T cells in TME [40]. Through co-culture model and in vivo model, we confirmed that efficacy of anti-TIGIT was CD8^+^T cells-dependent. Studies about anti-TIGIT also mentioned its’ ability to activate NK cells [41, 42]. Considering optimized NK cells by anti-HER2/Neu triggered ADCC, the ability of anti-TIGIT benefited NK cells might be masked [43]. Abolished CD226 inhibited the function of CD8^+^CD226^+^T cells and promoted tumor escape of immune surveillance in the presence of anti-TIGIT, indicated CD8^+^CD226^+^T cells might become an indicator and biomarker for evaluating anti-TIGIT. The mechanism by which TIGIT blockade leads to the coordinate upregulation of TBX21 and CD226 likely involves a multi-step rewiring of the tumor immune landscape. Recent evidence indicates that anti-TIGIT antibodies with an active Fc domain can engage Fcγreceptors on tumor-associated myeloid cells, enhancing their immunostimulatory capacity and altering the cytokine milieu [44]. In the context of concurrent anti-HER2 therapy, this creates a pro-inflammatory microenvironment that provides strong activation signals to CD8⁺ T cells, potentiating pathways such as PI3K-AKT-mTOR, a key inducer of the transcription factor TBX21 (T-bet) [39]. TBX21, a master regulator of type 1 immunity, then executes a transcriptional program that includes the upregulation of CD226, a co-stimulatory receptor critical for T cell function. This co-regulation of TBX21 and CD226 is a conserved feature of inflammatory Th1-like responses [45]. Furthermore, anti-TIGIT treatment concurrently prevents TIGIT from outcompeting CD226 for ligand binding, thereby releasing an extracellular brake on CD226 signaling [39]. The resultant synergy between increased CD226 expression and enhanced ligand engagement amplifies co-stimulatory signals, which, together with TBX21 activity, drives the robust IFN-γ production and cytotoxic function that underlies the efficacy of the combination therapy in HER2⁺ breast cancer. To conclude, our study offered a novel strategy to HER2^+^ breast cancer.

Limitation: We finally used AT3, a triple negative breast cell line from B6 strain, in our co-culture system considering OT-1 mice background. Tubo cell was a cell line harvested from Balb/c-NeuT mice, which showed mismatched MHC-I expression comparing with OT-1 CD8^+^T cells [46]. In order to obtain HER2 breast cancer cell line from B6 strain, we also try to convert MMTV/Neu mice to B6 background by crossing with B6 mice until F6. However, these crossed MMTV/Neu mice did not form tumor spontaneously after 1 year, while original MMTV/Neu mice form tumor spontaneously by 205 days (data not shown). This may because single oncogene cannot drive tumorigenesis in B6 strain. Although high ratio of CD8^+^CD226^+^T cells were induced by anti-TIGIT and associated with favorable OS and DFS in HER2⁺ breast cancer patients, further studies will need to validate the efficacy of anti-TIGIT and the relevance of CD226 for the efficacy of anti-TIGIT in real-world cohorts.

## Experimental section

### Patients and tumor samples

The 200-tissue microarray is built from patients diagnosed with HER2⁺ invasive breast carcinoma at Fudan University Shanghai Cancer Center (FUSCC). 28 HER2⁺ non-pCR patients were receiving standard neoadjuvant chemotherapy and Miller-Payne grade I-III. One patient was lost to follow-up; therefore, the final analysis included 27 patients. Before treatment samples were core needle biopsy (CNB) samples collected from 27 HER2⁺ invasive breast carcinoma before standard treatment, while post treatment samples were pathological slides after surgical dissection. HER2⁺ were defined as immunohistochemistry (IHC) HER2 3+ or IHC HER2 2+ with HER2 fluorescence in situ hybridization (FISH) amplification.

### Cell culture, lentiviral production, and cell line generation

Tubo (Fenghui, CL0719, China; RRID:CVCL_2A33) and AT3 (OriCell, M2-0901, China; RRID:CVCL_VR89) cells were obtained from FUSCC; these cells had been purchased in 2020 from Hunan Fenghui Biotechnology and OriCell, respectively. Both cell lines were cultured in DMEM (BasalMedia, L110KJ, China), 10%FBS (BI, 04-001-1 A, Israel), and 1% Penicillin-Streptomycin (BasalMedia, S110JV, China) at 37°C under 5% CO_2_. Cell lines in this study are available upon request. 600 μl Opti-MEM (Gibco, 31985070, America) mixture containing 4.5 μg psPAX2 (Addgene, 10878, America), 1.8 μg pMD2G (Addgene, 12259, America), 30 μl PEI (Sigma, 919012, America) and 6 μg targeted plasmid were used to transfect. Lentivirus were produced by HEK293T cells and filtered by 0.45 μM filter. For cell line Generation, cells were cultured with filtered lentivirus in the presence of 5 μg/ml polybrene (Sigma, 107689, America) for 8 hours and then sorted by flow cytometry with GFP. For selection, cells were cultured in media with 2 μg/ml blasticidin (InvivoGen, ant-bl-1, China). All cell lines were authenticated by STR profiling and were regularly tested to exclude mycoplasma contamination.

### Plasmids

pCDH-CMV-dOVA-EF1-BSD was designed followed by previous study [47]. pWPXL-Luciferase was purchased from Addgene.

### CD8^+^T isolation and stimulation

OT-1 CD8^+^T cells were harvested from spleen of OT-1 mice according to CD8a^+^T cell isolation kit (Miltenyi biotec, 130-104-075, Germany). Isolated CD8^+^T cells were plated in a 24-well plate (1 × 10^6^/well) using DMEM media, 10%FBS, 1%NEAA (BasalMedia, S220JV, China), 1‰β- Mercaptoethanol (Gibco, 21985023, America) and 1% Penicillin-Streptomycin. Dynabeads mouse T-activator CD3/CD28 beads (ThermoFisher, 11452D, America) and 30U mIL-2 (PeproTech, 212-12, America) were used to activate CD8^+^T cells followed by manufacture’s protocol.

### Tumor-CD8^+^T cell co-culture assay

AT3-GFP-OVA cells were pretreated with 0.02 nM SIINFEKL peptide (Sigma, 138831-86-4, America) and 2 ng/mg IFN-γ (PeproTech, 301-02, America) for 12 h before use. For tumor MHC-I expression, 1 × 10^6^ AT3-GFP-OVA cells were incubated with anti-mouse H2K^b^ (Biolegend, 141605, America). For co-culture, AT3-GFP-OVA cells were co-cultured with activated OT-1 CD8^+^T cells (AT3-GFP-OVA: OT-1 CD8^+^T = 8000:40000) in the presence of 0.75 μg SINFEKL peptide and 30U mIL-2 for 24 hours in a 96-well plate. Cells were incubated with appropriate antibodies befor flow cytometry. Killing rate of tumor cells equals to FSV780^+^GFP^+^ cells divided by co-culture cells.

### Flow cytometry analysis

To obtain single cell suspension, we applied digestion solution (1:20 Collagenase I, 1:20 Collagenase III, 1:200 hyaluronidase and 1:1000 DNase I in DMEM media). After minicing, fragments were placed in a 15 ml tube with 10 ml digestion solution and incubated in 37°C for 30 minutes on a rotator followed by red cell lysis with ACK buffer (8.29 g NH_4_Cl, 1 g KHCO_3_ and 1 mM EDTA with pH 7.2–7.4). Single cell suspensions were obtained by a cell filter and were stained with appropriate antibodies after blocking. Cell suspensions were analyzed by CytoFLEX (Beckman). Data were analyzed using FlowJo v10.8.1 and CytExpert v2.3 software.

### scRNA-data processing

Low quality of cells was assessed using R package Seurat (v4.3.0). Briefly, features below 200 and above 8000, counts below 150,000, percentage of red cell gene above 2 and percentage of mitochondrial genes above 20 were filtered. NormalizeData function was used to remove batch effect. FindVariableFetaures function was used to pick 3000 highly variable genes and cell clusters in each sample were identified by examining the top 50 principal components (PCs) across highly variable genes (HVGs) using RunPCA function. Next, doublet cells were depleted using DoubletFinder (2.0.3). Finally, harmony (0.1.1) was used to integrate samples after quality control.

### Cell clustering and annotation

FindNeighbors and FindClusters function were used to calculate top 50 PCs. The dimensionality in this study was reduced using UMAP. We identify cell clusters by FindAllMarker function and annotate clusters using SingleR (1.8.1) automatically. Modified annotation were used by published scRNA-data [48–50]. We performed second round clustering to divide subpopulations of malignant cells and T/NK cells to further characterize major cell types.

### Cell-cell interaction

To investigate cell-cell interaction among malignant cells and immune cells, we analyzed L-R pairs using R toolkit CellChat (1.4.0). To identify potential L-R pairs, we used ranknet function to rank signaling from malignant cells to re-clustered T/NK cells. Significant L-R pairs were *P* < 0.05 using Wilcoxon test.

### Enrichment network analysis

R toolkit AuCell (1.16.0) were used to assess enrichment scores in different groups. Gene sets of HALLMARK and C2 were used to perform enrichment analysis and downloaded from Molecular Signatures Database (MSigDB v.7.5). Significant scores were *P* < 0.05 using Kruksal–Wallis test. R toolkit DoRothEA (1.6.0) were used to calculate transcriptional factors.

### Dataset latent semantic indexing (LSI) space match

For project scRNA-seq data into given LSI space, we applied R package ProjectLSI (0.1.0) to match dataset.

### Mouse models

All mice in this study were bred in a pathogen-free environment according to animal facility guideline in FUSCC. MMTV/Neu mice (FVB/N-Tg(MMTVneu)202Mul/J, Stock No. 002376) were purchased from Jackson Laboratory. OT-1 mice (C57BL/6-Tg(TrcaTrcb)Smoc) were purchased from Shanghai Model Organisms Center. Balb/c mice were purchased from Shanghai Jihui Lab-animal.

For HER2 non-sensitive mouse model, 5 × 10^5^ Tubo were implant in Balb/c mice at fourth mammary pad at day0 in PD group, day 14 in ctrl and SD group. In PD group, mice receiving 5 mg/kg anti-Neu (BioXcell, BE0277, America; RRID:AB_2687800) by intraperitoneal injection at day4,7 and 10; 10 mg/kg anti-Neu at day14, 18 and 21; 20 mg/kg anti-Neu at day 25 and 29. PD was defined as when tumor volume decreased at day 25 and increased again at day 29. In ctrl and SD group, 20 mg/kg isotype IgG (BioXcell, BE0085, America; RRID:AB_1107771) and anti-Neu were injected by intraperitoneal injection at day 4, 7, 10, 14, 18, 21, 25 and 29 in ctrl group and SD group respectively.

For anti-TIGIT treatment, mice received 20 mg/kg Isotype IgG, 10 mg/kg anti-Neu+10 mg/kg IgG and 10 mg/kg anti-Neu+10 mg/kg anti-TIGIT (Biolegend, 622204, America; RRID:AB_2814473) by intraperitoneal injection at day6, 9, 13, 16 and 20 in ctrl, anti-Neu and combined group respectively.

For CD8^+^T depletion treatment, mice receiving anti-CD8a (BioXcell, BE0117, America; RRID:AB_10950145) at day0, 7, 13, 19 and 25. This regimen was applied to all mice in the designated experiments. 5 × 10^5^ Tubo were implant in Balb/c mice at fourth mammary pad at day7; 10 mg/kg isotype IgG, 10 mg/kg anti-Neu, 10 mg/kg anti-Neu and 10 mg/kg anti-TIGIT were injected by intraperitoneal injection at day13, 16, 19, 22 and 25 in ctrl group, anti-Neu group and combined group respectively.

For adoptive infusion, mice underwent the same anti-CD8a depletion schedule. Subsequently, 5 × 10^5^ CFSE-labeled CD8^+^T cells were injected by tail vein at day 16 and day23. Tumor treatment schedules remained identical to the CD8 depletion treatment.

### Integration of TCGA and METABRIC dataset

We choose HER2^+^ breast cancer patients according to clinical information. Finally, 194 HER2^+^ breast cancer patients after removal of batch effect were used in further survival analysis.

### Survival analysis

Kaplan-Meier survival curves were plotted using R toolkit survminer (0.4.9). The cutoff of high and low TIGIT^+^CD8^+^ T cells and CD8^+^CD226^+^T cells were defined by median numbers under 200 μm microscope landscape. TIGIT^+^CD8^+^T signature scores were calculated by the product of *CD8A*, *CD8B* and *TIGIT* divided by the mRNA level of *CD3D*, *CD3E* and *CD3G* [21].

### Tissue multiplex immunofluorescence staining (IHF)

Initially, we dried slides at 60 °C for 1 h and deparaffinized slides in xylene. After pretreated, we block unspecific binding sites using 10% goat serum (Sangon Biotech, E510009-0100, China). Mouse-anti-CD8a (1:500, Proteintech, 66868-1-Ig, China; RRID:AB_2882205), rabbit-anti-TIGIT (1:500, CST, 99567T, America; RRID: AB_3718000), rabbit-anti-CD226 (1:200, CST, 66631T, America; RRID: AB_3718001) were applied overnight at 4 °C. Goat anti-rabbit IgG (ORIGENE, PV6001, China; RRID:AB_2864333) and Goat anti-mouse (ORIGENE, PV6002, China; RRID:AB_2864334) were applied 1 h at room temperature, followed by mounting with DAPI-containing mounting medium (Yeasen, 36308ES11, China). Fluorescence images were captured by Leica microscope (Leica, DMI6000B, Germany).

### Tissue immunohistochemistry

Slides were dried, deparaffinized and pretreated before blocking. 10% goat serum was used to block unspecific binding sites. Rabbit-anti-CD155 (1:500, abcam, ab267788, America; RRID:AB_3669016), rabbit-anti-CD112 (1:200, CST, 95333S, America; RRID: AB_3718002) were applied overnight at 4 °C. DAB staining was followed manufacturer’s instruction (Gene Tech, GK600510, China). Images were captured by Olympus (Olympus, CX23, America). Expression levels of CD112 and CD155 were independently evaluated by two pathologists. Scoring criteria were defined as follows: 3+: >10% of tumor cells showing circumferential, intense, and complete membranous staining. 2+: >10% of tumor cells showing weak to moderate membranous staining. 1+: >10% of tumor cells showing incomplete, faint, or barely visible membranous staining. neg: No staining, or ≤10% of tumor cells with barely visible incomplete staining. For analytical purposes, scores of 1+ or neg were classified as “low or negative” expression, while scores of 2+ or 3+ were classified as “high” expression.

### Statistical analysis

Single cell RNA-seq data was carried out with R software (4.1.3). The pair and unpair Student’s *t* test and ANOVA test were used to performed in Prism (8.0.1). Non parametric data were analyzed by Mann–Whitney U test or Kruksal–Wallis test in SPSS 20 software. Statistical comparisons for paired IHC scores were performed using the Wilcoxon signed-rank test in SPSS 20 software. Significant thresholds were set at *P < 0.05; **P < 0.01; ***P < 0.001; ****P < 0.0001.

## Supplementary information

Supplementary figure-figure legend

Supplementary figure-FigureS1

Supplementary figure-FigureS2

Supplementary figure-FigureS3

Supplementary figure-FigureS4

Supplementary figure-FigureS5

Supplementary figure-FigureS6

Supplementary figure-FigureS7

Supplementary figure-FigureS8

Supplementary figure-FigureS9

## Data Availability

Any additional information required to reanalyze the data reported in this paper is available from the lead contact upon request.
