# Supplementary material for: Restoring the CD226 in CD8+T cells overcomes TIGIT-refractory immunity in HER2+ breast cancer
Source: Cell Death Dis. 2026 Jul 13;17(1):868. doi: 10.1038/s41419-026-09004-5 (PMC13642357; doi:10.1038/s41419-026-09004-5)
Supplement: Supplementary file 1 [file 41419_2026_9004_MOESM1_ESM.docx]

**Fig. S1 |** (a) Cell type annotation of single-cell RNA sequencing data from 10 HER2^+^ breast cancer patients. (b) Re-clustering and annotation of the T cell compartment in the 10 HER2^+^ breast cancer patients. (c) Cell type annotation of single-cell RNA sequencing data from a rtTA-HER2Δ16 spontaneous HER2^+^ mouse tumor model. (d) Re-clustering and annotation of the T cell compartment in the rtTA-HER2Δ16 spontaneous HER2^+^ mouse tumor model. (e) Principal component analysis (PCA) plot of HER2^+^ breast cancer samples from the TCGA and METABRIC cohorts prior to batch effect removal. (f) PCA plot of integrated HER2^+^ breast cancer data from TCGA and METABRIC cohorts after batch effect correction. (g) Kaplan–Meier survival curves of HER2^+^ breast cancer patients from TCGA and METABRIC dataset, stratified by TIGIT expression. Significance levels denote statistically differential expression between groups (ns indicates no statistically significant difference).

**Fig. S2 |** (a) Confocal microscopy image showing localization of the Neu protein (red) in the HER2^+^ murine breast cancer cell line Tubo. Nuclei are counterstained with DAPI (blue). (b) Flow cytometry gating strategy for immune cell identification across three experimental groups.

After excluding debris and dead cells by gating on viability dye-negative populations (FVS780), live cells were selected. CD45-positive immune cells were subsequently gated from the live cell population. Panels from top to bottom correspond to the Control (Ctrl), SD, and PD groups, respectively. (c) Proportion of tumor-infiltrating CD8⁺PD-1⁺ T cells across three groups. (Left to right: Ctrl, SD, and PD). Data are derived from flow cytometric analysis of dissociated tumor tissues. (d) Statistical analysis of tumor-infiltrating CD8⁺PD-1⁺ T cell proportions across three experimental groups. ns indicates no statistically significant difference. (e) Proportion of tumor-infiltrating TIGIT⁺NK cells across three groups. (Left to right: Ctrl, SD, and PD). Data are derived from flow cytometric analysis of dissociated tumor tissues. (f) Statistical analysis of tumor-infiltrating TIGIT⁺NK cell proportions across three experimental groups. ns indicates no statistically significant difference.

**Fig. S3 |** (a) CD112 IHC staining intensity and response categories from FUSCC (n=200). (Top panel) Representative IHC images of CD112 expression in tumor tissue samples, categorized as CD112^neg^ (-), CD112^lo^ (+), CD112^int^ (2+), and CD112^hi^ (3+) from left to right. Staining intensity was evaluated according to the criteria detailed in the Materials and Methods section. (Bottom panel) Classification of CD112 dynamic changes in response to treatment: CD112 stable or downregulated (no change or decrease in IHC staining intensity post-treatment) and CD112 upregulated (increase in staining intensity post-treatment). Scale bar: 30 μm. (b) CD112 IHC staining changes in 27 HER2^+^ breast cancer patients with non-pCR following neoadjuvant therapy, whereas asterisks denote a significant difference (***p < 0.001). (c) CD155 IHC staining intensity and response categories from FUSCC (n=200). (Top panel) Representative IHC images of CD155 expression in tumor tissue samples, categorized as CD155^neg^ (-), CD155^lo^ (+), CD155^int^ (2+), and CD155^hi^ (3+) from left to right. Staining intensity was evaluated according to the criteria detailed in the Materials and Methods section. (Bottom panel) Classification of CD155 dynamic changes in response to treatment: CD155 stable or downregulated (no change or decrease in IHC staining intensity post-treatment) and CD155 upregulated (increase in staining intensity post-treatment). Scale bar: 30 μm. (d) CD155 IHC staining changes in 27 HER2^+^ breast cancer patients with non-pCR following neoadjuvant therapy. No significant difference was denoted by ns (p = 0.683). (e) Representative images of CD155 IHC staining from FUSCC (n=200). Left: classified as low or negative CD155 expression. Right: classified as high CD155 expression. (f) Overall survival curves of 200 HER2^+^ breast cancer patients from FUSCC, stratified by CD155 expression (***p < 0.001). (g) Disease free survival curves of 200 HER2^+^ breast cancer patients from FUSCC, stratified by CD155 expression (**p < 0.01).

**Fig. S4 |** (a) Tumor images across four experimental groups. (Left, top to bottom: Ctrl, Anti-Neu, Anti-TIGIT and Combined). Tumor proliferation curve showed in four groups (Right, day 20 mean volume: Ctrl:867.98 mm^3^, Anti-Neu:275.94 mm^3^, Anti-TIGIT:215.63 mm^3^, Combined:93.27 mm^3^), whereas asterisks denote a significant difference (*p < 0.05, **p < 0.01). (b) Cell type annotation of single-cell RNA sequencing data from the Tubo mouse tumor model treated with anti-HER2/Neu and anti-TIGIT therapy. (c) UMAP plot of epithelial cell subpopulations from the Tubo mouse tumor model following anti-HER2/Neu and anti-TIGIT combination therapy. (d) AUC scores of scRNA-seq data from anti-Neu-resistant subclones treated with Control, anti-Neu monotherapy, or Combination therapy. (e) T cell re-clustering and annotation from scRNA-seq data of the Tubo mouse tumor model treated with anti-HER2/Neu and anti-TIGIT combination therapy. (f) Proportional changes in T cell subsets of single-cell RNA sequencing data from the Tubo mouse tumor model across Control, Anti-Neu, and Combined treatment groups. (g) UMAP plot of scRNA-seq data from the MM3MG-HER2Δ16 mouse tumor model treated with anti-HER2 therapy. (h) Cell type annotation of scRNA-seq data from the MM3MG-HER2Δ16 mouse tumor model treated with anti-HER2 therapy.

**Fig. S5 |** (a) Integration and classification of epithelial cells from HER2Δ16-driven tumor models. Epithelial cells from scRNA-seq data of the MM3MG-HER2Δ16 mouse tumor model were projected onto the Tubo mouse tumor model using the ProjectLSI package. (b) Based on their positions in the projected latent space, epithelial cells were classified into 4D5-resistant (insensitive to anti-HER2 antibody therapy) and sensitive subsets. (c) UMAP plot of T cell re-clustering from scRNA-seq data in the MM3MG-HER2Δ16 mouse tumor model treated with anti-HER2 therapy. (d) T cell re-clustering and annotation from scRNA-seq data of the MM3MG-HER2Δ16 mouse tumor model treated with anti-HER2 therapy. (e) UMAP plot of *Pdcd1* T cell re-clustering from scRNA-seq data in the MM3MG-HER2Δ16 mouse tumor model treated with anti-HER2 therapy. (f) *Pdcd1* T cell re-clustering and annotation from scRNA-seq data of the MM3MG-HER2Δ16 mouse tumor model treated with anti-HER2 therapy.

**Fig. S6 |** (a) Differential intercellular communication between Anti-Neu group and Control groups in Tubo mouse tumor model. Signaling edges colored in red represent interactions stronger in the Control group, while green edges indicate interactions enhanced in the Anti-Neu group. Absence of significant enhancement in specific signals (CD226, MHC-I, and IFN-II response) in the Anti-Neu monotherapy group. (b) Differential intercellular communication between Anti-4D5 group and Control groups in MM3MG-HER2Δ16 mouse tumor model. Signaling edges colored in red represent interactions stronger in the Control group, while green edges indicate interactions enhanced in the Anti-4D5 group. Absence of significant enhancement in specific signals (CD226, MHC-I, and IFN-II response) in the Anti-4D5 monotherapy group. (c) Expression of *Pdcd1* in CD8⁺ T cell subsets across three treatment groups in Tubo mouse tumor model. (top to bottom: *Cd8 Ccr7* T cells, *Cd8 Gzmk* T cells, and *Cd8 Pdcd1* T cells). scRNA-seq shows normalized gene expression levels of *Pdcd1*. Data are represented for the Control group (red), anti-Neu monotherapy group (green), and Combination therapy group (blue). ns indicates no statistically significant difference. (d) Expression of *Pdcd1* in CD8⁺ T cell subsets across three treatment groups in MM3MG-HER2Δ16 mouse tumor model. (Top to bottom: *Cd8 Ccr7* T cells, *Cd8 Gzmk* T cells, and *Cd8 Pdcd1* T cells). scRNA-seq shows normalized gene expression levels of *Pdcd1*. Data are represented for the Control group (red) and anti-4D5 monotherapy group (green). ns indicates no statistically significant difference. (e) Ranking of ligand-receptor pair strengths based on interaction scores in the Control group (top). Cell communication of MHC I signaling between T/NK cell subsets and tumor subclones. Edge thickness represents the inferred interaction strength, and arrows indicate the direction of signaling from sender to receiver cells. No signaling activity for the H2d1-Cd8b1 (middle) or H2k1-Cd8b1 (bottom) pairs was detected between NK cells and tumor cells. Analysis performed using CellChat package. (f) Ranking of ligand-receptor pair strengths based on interaction scores in the Anti-Neu group (top). Cell communication of MHC I signaling between T/NK cell subsets and tumor subclones. Edge thickness represents the inferred interaction strength, and arrows indicate the direction of signaling from sender to receiver cells. No signaling activity for the H2d1-Cd8a (middle) or H2d1-Cd8b1 (bottom) pairs was detected between NK cells and tumor cells. Analysis performed using CellChat package. (g) Ranking of ligand-receptor pair strengths based on interaction scores in the Combined group (top). Cell communication of MHC I signaling between T/NK cell subsets and tumor subclones. Edge thickness represents the inferred interaction strength, and arrows indicate the direction of signaling from sender to receiver cells. No signaling activity for the H2d1-Cd8b1 (middle) or H2k1-Cd8b1 (bottom) pairs was detected between NK cells and tumor cells. Analysis performed using CellChat package.

**Fig. S7 |** (a) AUC scores of T cell activation involved in immune response in CD8⁺ T cell subsets across three treatment conditions. scRNA-seq analysis of CD8⁺ T cell subsets (left to right: *Cd8 Ccr7* T cells, *Cd8 Gzmk* T cells, and *Cd8 Pdcd1* T cells) treated with Control, anti-Neu monotherapy, or Combination therapy. (b) AUC scores of IFN-II production in CD8⁺ T cell subsets across three treatment conditions. scRNA-seq analysis of CD8⁺ T cell subsets (left to right: *Cd8 Ccr7* T cells, *Cd8 Gzmk* T cells, and *Cd8 Pdcd1* T cells) treated with Control, anti-Neu monotherapy, or Combination therapy.

**Fig. S8 |** (a) Flow cytometric analysis of GFP and SIINFEKL expression in AT3-GFP-OVA engineered cells. Left to right: AT3-pCDH and AT3-GFP-OVA cells. X-axis exhibited GFP fluorescence intensity while y-axis showed SIINFEKL peptide levels. (b) Flow cytometry gating strategy for analysis of tumor cell killing in AT3-GFP-OVA and OT-1 CD8^+^T cells co-culture system. Left:control group. Right: anti-TIGIT group. (c) Flow cytometric gating strategy for analysis of CD8⁺CD226⁺T cell proportion in AT3-GFP-OVA and OT-1 CD8^+^T cells co-culture system. After excluding debris and dead cells by gating on viability dye-negative populations (FVS780), live cells were selected. CD3-positive T cells were subsequently gated from the live cell population. Top to bottom: control group and anti-TIGIT group. (d) Flow cytometry gating strategy for detection of MHC class I (H2-K^b^) expression on tumor cells in AT3-GFP-OVA and OT-1 CD8^+^T cells co-culture system. After excluding debris and dead cells by gating on viability dye-negative populations (FVS780), live cells were selected. Tumor cells were subsequently gated from the live cell population via GFP expression. Top to bottom: control group and anti-TIGIT group. (e) CD8a IHC staining of CD8⁺T cells in tumor tissues from the CD8⁺T cell depletion experiment. Left: Control group. Right: CD8⁺T cell-depleted group. Scale bar: 30 μm. (f) Statistical analysis of CD8⁺T cell numbers across in two groups. Mean numbers: Ctrl:18.20, CD8⁺T cell-depletion:2.00 (*p < 0.05). (g) Preparation of CD8⁺T cells for adoptive transfer via CD8a magnetic bead sorting and CFSE labeling.

**Fig. S9 |** (a) Flow cytometry gating strategy for analysis of tumor cell killing in AT3-GFP-OVA and OT-1 CD8^+^T cells co-culture system. Top to bottom: control group, anti-TIGIT group and anti-TIGIT+anti-CD226 group. (b) Flow cytometric gating strategy for analysis of CD8⁺CD226⁺T cell proportion in AT3-GFP-OVA and OT-1 CD8^+^T cells co-culture system. After excluding debris and dead cells by gating on viability dye-negative populations (FVS780), live cells were selected. CD8-positive T cells were subsequently gated from the live cell population. Top to bottom: control group, anti-TIGIT group and anti-TIGIT+anti-CD226 group. (c) Flow cytometry gating strategy for detection of MHC class I (H2-K^b^) expression on tumor cells in AT3-GFP-OVA and OT-1 CD8^+^T cells co-culture system. After excluding debris and dead cells by gating on viability dye-negative populations (FVS780), live cells were selected. Tumor cells were subsequently gated from the live cell population via GFP expression. Top to bottom: control group, anti-TIGIT group and anti-TIGIT+anti-CD226 group. (d) UMAP plot of re-clustered tumor-infiltrating CD8⁺ T cells, with cells colored by the normalized expression level of TBX21. Cells are stratified into two meta-clusters based on CD226 expression (top). Statistical analysis of TBX21 expression across in two groups. Mean expression: Cd226 low:0.04, Cd226 high:0.25 (****p < 0.0001) (bottom).
